# Ciprofloxacin-Resistant *Shigella sonnei* among Men Who Have Sex with Men, Canada, 2010

**DOI:** 10.3201/eid1709.102034

**Published:** 2011-09

**Authors:** Christiane Gaudreau, Ruwan Ratnayake, Pierre A. Pilon, Simon Gagnon, Michel Roger, Simon Lévesque

**Affiliations:** Author affiliations: Centre Hospitalier de l’Université de Montréal–Hôpital Saint-Luc, Montreal, Québec, Canada (C. Gaudreau);; Université de Montréal, Montreal (C. Gaudreau, P.A. Pilon, S. Gagnon, M. Roger);; Agence de la Santé et des Services Sociaux de Montréal-Santé Publique, Montreal (R. Ratnayake, P.A. Pilon);; Public Health Agency of Canada, Ottawa, Ontario, Canada (R. Ratnayake);; Centre Hospitalier de l’Université de Montréal–Hôpital Notre-Dame, Montreal (S. Gagnon, M. Roger);; Laboratoire de Santé Publique du Québec/Institut National de Santé Publique du Québec, Sainte-Anne-de-Bellevue, Quebec (S. Lévesque)

**Keywords:** bacteria, Shigella sonnei, emergence, ciprofloxacin, antimicrobial resistance, men, Quebec, MSM, sexually transmitted infection, STI, anal sex, dispatch

## Abstract

In 2010, we observed isolates with matching pulsed-field gel electrophoresis patterns from 13 cases of ciprofloxacin-resistant *Shigella sonnei* in Montréal. We report on the emergence of this resistance type and a study of resistance mechanisms. The investigation suggested local transmission among men who have sex with men associated with sex venues.

*Shigella* spp. are enteropathogen bacteria that are transmitted person-to-person and require a low infectious inoculum (*1*). Fluoroquinolones are among the first-choice antimicrobial drugs for treatment of *Shigella* spp. infections in adults (*1*), but resistance to these agents has been documented, primarily in Asia (*2*). Among men who have sex with men (MSM), *Shigella* spp. infection is, in most cases, sexually transmitted, and clusters are regularly reported (*3**–**5*). We investigated an outbreak of ciprofloxacin-resistant *Shigella sonnei* among MSM and studied its resistance mechanisms.

## The Study

Laboratories report shigellosis to the Montreal public health department (Quebéc, Canada). When a cluster is suspected, isolates are sent to the provincial laboratory to conduct pulsed-field gel electrophoresis (PFGE) to identify links between patients.

In July 2010, microbiology services at the Hôpital Saint-Luc alerted the public health department to *S. sonnei* resistant to ciprofloxacin and trimethoprim/sulfamethoxazole and susceptible to ampicillin. The *S. sonnei* had been isolated 2 days apart from stool cultures of 2 HIV-positive MSM.

Public health officials sent a notice to physicians, clinics, and laboratories in Montréal to report the presence of ciprofloxacin-resistant *S. sonnei* among MSM and to describe the antimicrobial treatment with ampicillin or azithromycin, procedures for case reporting, and preventive measures (*6*). Confirmed cases were defined as infection by *S. sonnei* with resistance to ciprofloxacin and trimethoprim/sulfamethoxazole and susceptibility to ampicillin (later specified as pulsovar 72). Probable cases were defined as infection by *S. sonnei* with a resistance profile identical to that of confirmed cases but where PFGE was not conducted.

**Figure 1:**
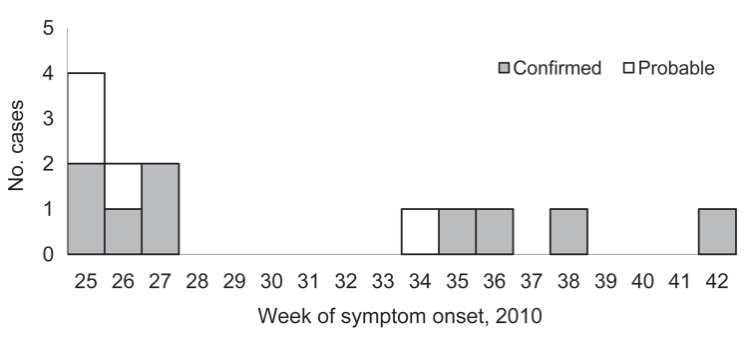
Confirmed and probable cases of ciprofloxacin-resistant *Shigella sonnei* infection, by week of onset, Montréal, Québec, Canada, June–October 2010.

Retrospective searching of the notifiable disease database found ciprofloxacin-resistant *S. sonnei* with a different PFGE pattern that had been isolated in February 2010 from a female patient who had traveled to a country where shigellosis is highly prevalent. Hence, this case was not from this outbreak. The provincial laboratory searched their records to identify cases elsewhere in Québec. During June–October 2010, nine confirmed cases and 4 probable cases were identified in Montréal and the surrounding regions (Table 1). Most patients had an onset date from the end of June to mid-July 2010 (Figure 1). All 13 patients were interviewed. Most patients were men (11/13; 85%) with a mean age of 40 years (range 20–65 years). All male patients were MSM, and 4 (36%) of 11 reported being HIV positive. Travel to a European country during August 2010 was mentioned by 1 MSM patient. Eight (73%) of 11 MSM patients mentioned participation in anal sex or contact during the exposure period. The use of sex venues was indicated by 4/11 MSM patients, and 3 mentioned a common sex venue. In addition, 1 other MSM patient reported that his sex partners frequented the common sex venue. This suggests that unprotected anal sex, associated with local sex venues, was the primary mode of transmission.

**Table 1:** Characteristics of 13 cases of ciprofloxacin-resistant *Shigella sonnei* and results of susceptibility testing, Montreal, Québec, Canada, June–October 2010*

Patient ID	Patient age, y/sex	Stool sample date	Patient’s signs and symptoms	Antimicrobial agent			PFGE group
Amp	TMP/SMX	Cip
1	52/M	Jun 24	Diarrhea	S	R	R	72
2	48/M	Jun 26	Nausea, vomiting, diarrhea, blood in stools, abdominal pain	S	R	R	NT
3	22/M	Jun 28	Nausea, diarrhea, abdominal pain, fever	S	R	R	NT
4	38/M	Jul 5	Nausea, vomiting, diarrhea, abdominal pain, fever, fatigue	S	R	R	72
5	52/M	Jul 4	Diarrhea, abdominal pain, fever	S	R	R	72
6	32/M	Jul 14	Diarrhea	S	R	R	NT
7	50/F	Jul 11	Nausea, diarrhea, blood in stools, abdominal pain, fever	S	R	R	72
8	45/F	Jul 14	Diarrhea, blood in stools, abdominal pain	S	R	R	72
9	25/M	Aug 30	Diarrhea, blood in stools, abdominal pain	S	R	R	NT
10	50/M	Sep 5	Vomiting, diarrhea, abdominal pain, fever	S	R	R	72
11	65/M	Sep 8	Nausea, vomiting, diarrhea, abdominal pain	S	R	R	72
12	38/M	Sep 23	Diarrhea	S	R	R	72
13	20/M	Oct 22	Diarrhea, blood in stools, abdominal pain, fever	S	R	R	72

*ID, identification; Amp, ampicillin; TMX-SMX, trimethoprim/sulfamethoxazole; Cip, ciprofloxacin; PFGE, pulsed-field gel electrophoresis; S, sensitive; R, resistant; NT, not typed.

Two female patients (45 and 50 years of age) were reported. *S. sonnei* was detected in a food sample from a restaurant where 1 female patient ate during the exposure period, but the isolate did not match the outbreak PFGE pattern and was not related to any known human patients. No epidemiologic links between the female and male patients could be identified.

The public health interventions included a weekly analysis of incident shigellosis infections, resistance profiles, and risk factors. Given the preponderance of infections among MSM visiting sex venues, kits of condoms, soap, and information on prevention were distributed at sex venues in August 2010 (*3**,**6*). Community-based organizations that work with MSM living with HIV/AIDS were contacted to disseminate information on preventive measures. As a potential effect, few cases were declared in September, although sporadic cases continued to appear until October 2010.

The resistance profile investigation identified 14 *S. sonnei* isolates from 13 patients by using commercial biochemical kit tests. Identification of *S. sonnei* from 9 patients was confirmed at the provincial laboratory. Antimicrobial susceptibility testing was done by agar dilution or disk diffusion method (*7*), Vitek 2 (bioMérieux, Marcy l’Étoile, France), or Etest (AB Biodisk, Solna, Sweden) (ampicillin, trimethoprim/sulfamethoxazole, and ciprofloxacin) for 14 isolates and by Etest (AB Biodisk) (azithromycin, cefotaxime, and tetracycline) and with nalidixic acid (30-μg disk) for 7 or 8 isolates. The susceptibility of *S. sonnei* isolates to antimicrobial agents is reported in Table 1 and Table 2.

**Table 2:** Antimicrobial drug susceptibility of *Shigella sonnei* isolates to 7 agents, Montreal, Québec, Canada, 2010*

Antimicrobial agent	No. isolates tested	MIC	Interpretation
Ampicillin	11	2–8 mg/L	S
TMP/SMX	11	>32 mg/L	R
Ciprofloxacin	11	>4–16 mg/L	R
Azithromycin	8	8–16 mg/L	NA
Cefotaxime	8	0.06–0.125 mg/L	S
Tetracycline	7	256–>256 mg/L	R
Nalidixic acid	8	6 mm†	R

*The susceptibility and resistance breakpoints were those for *Enterobacteriaceae,* except for azithromycin, for which these breakpoints are not available (*7*). TMP/SMX, trimethoprim/sulfamethoxazole; S, susceptible; R, resistant; NA, not available. †By disk diffusion method (*7*).

**Figure 2:**
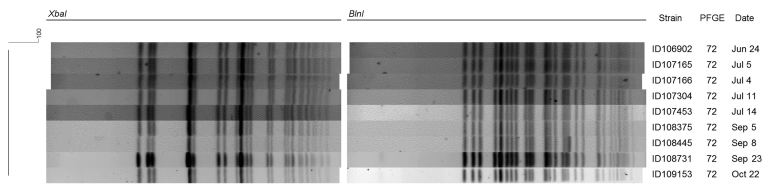
Digestion pattern of ciprofloxacin-resistant *Shigella sonnei* isolated from 9 patients for *Xba*I and *Bln*I, Montréal, Québec, Canada, June–October 2010. PFGE, pulsed-field gel electrophoresis.

PFGE was done by the provincial laboratory according to international standards set by the US Centers for Disease Control and Prevention (*8*). The *Xba*I and *Bln*I patterns were interpreted using the standards of Tenover et al. (*9*). The *Salmonella enterica* serotype Braenderup strain (H9812) was used as the size marker in each gel (*10*). Band position tolerances and optimization values of 1% were used for all analyses. Similarity coefficient was obtained within BioNumerics (www.applied-maths.com/bionumerics/bionumerics.htm) by calculating Dice coefficients. Cluster analysis was done by using with the unweighted pair group method with arithmetic averages. The *S. sonnei* isolates from 9 patients for whom typing was done were indistinguishable for the 2 enzymes (Figure 2). PulseNet Canada accession numbers for the isolate from our study are SSOXAI.0067 and SSOBNI.0040 for the *Xba*I and *Bln*I patterns, respectively.

For the study of the mechanisms of drug resistance, bacterial DNA was extracted using MasterPure Complete DNA Purification Kit (Epicenter Biotechnologies, Madison, WI, USA). The *gyrA* and *parC* genes were analyzed by direct DNA sequencing procedures as described (*11*) on an ABI Prism Genetic Analyzer 3130xl (Applied Biosystems, Foster City, CA, USA). The DNA sequences were converted into amino acid sequences by using the EMBOSS Transeq tool (European Molecular Biology–European Bioinformatics Institute), aligned by using ClustalW (DNAStar, Madison, WI, USA), and compared with that of the reference quinolone-susceptible strain (GenBank accession no. NC_008258). The 8 *S. sonnei* strains from 7 patients harbored the same nonsynonymous substitutions in comparison with the quinolone-susceptible reference strain: S83L and D87G for *gyrA* and S80I for *parC*. These amino acid substitutions have been previously associated with ciprofloxacin resistance in *Escherichia coli* (*11*) and in *S. dysenteriae*, *S. flexneri*, and *S. boydii* (*2*) but not in *S. sonnei* isolates.

Blood in stools or fever was reported by 9 (69%) of 13 patients (Table 1). Of the known treatment outcomes, 2 of the 4 patients treated with oral ampicillin had a negative stool culture 48 hours and 72 hours after completion. One of the 2 patients treated with oral amoxicillin experienced a clinical and microbiologic treatment failure 48 hours after completion, but a clinical and microbiologic cure was achieved after treatment with oral azithromycin. Two other patients were treated with azithromycin and 1 other with ciprofloxacin.

## Conclusions

Sporadic ciprofloxacin-resistant *Shigella* has been infrequently documented (*2**,**12**–**14*). In India, ciprofloxacin-resistant *S. dysenteriae*, *S. flexneri,* and *S. boydii* have been isolated since 2002, and their fluoroquinolone-resistant mechanisms have been determined (*2*). Ciprofloxacin-resistant *Shigella* remains rare and was found among 0.2% of isolates in the United States (2000–2009) (*12*) and 0.5% of isolates in Canada (1997–2000) (*13*). In the United States, 10 ciprofloxacin-resistant *Shigella* spp. isolates (6 *S. flexneri*, 3 *S. sonnei*, and 1 *Shigella* spp.) were documented by the National Antimicrobial Resistance Monitoring System (2000–2009) (*12*). In New York, NY, in 2006, ciprofloxacin resistance was detected among 4 *S. sonnei* and 1 *S. flexneri* acquired locally (*14*). In 2010 in South Carolina, ciprofloxacin-resistant *Shigella flexneri* 2a was isolated from 3 patients (*15*).

We report the suspected transmission of ciprofloxacin-resistant *S. sonnei,* among MSM in Montreal, Québec. Some authors suggest the antimicrobial drug treatment of all patients infected with *Shigella* spp (*1*), but others disagree with this recommendation (*14*). It is essential that physicians request bacterial stool cultures when *Shigella* spp. enteric infection is suspected in MSM even without blood in stools or fever.
